# Esophageal pressure monitoring and ECCO_2_R: a personalized approach to ultraprotective mechanical ventilation in ARDS patients

**DOI:** 10.4103/mgr.MEDGASRES-D-25-00049

**Published:** 2026-04-11

**Authors:** Sergio Lassola, Silvia De Rosa, Francesco Alessandri, Denise Battaglini, Marcus J Schultz

**Affiliations:** Santa Chiara Hospital of Trento, Anesthesia and Intensive Care 1, Trento, Italy; Centre for Medical Sciences-CISMed, University of Trento, Trento, Italy; Intensive Care Unit, Department of General, Specialistic Surgery, “Sapienza” University of Rome, Rome, Italy; Azienda Ospedaliera-Universitaria Policlinico Umberto I, Rome, Italy; Anesthesia and Intensive Care, IRCCS Ospedale Policlinico San Martino, Genova, Italy; Department of Surgical Sciences and Integrated Diagnostics (DISC), University of Genova, Genova, Italy; Department of Intensive Care, Amsterdam University Medical Centers, Amsterdam, The Netherlands; Department of Anesthesia, General Intensive Care and Pain Management, Medical University Wien, Vienna, Austria; Nuffield Department of Medicine, University of Oxford, Oxford, UK; Mahidol-Oxford Research Unit (MORU), Mahidol University, Bangkok, Thailand

Mechanical ventilation (MV) is a fundamental intervention in critical care, offering essential life-saving support for patients experiencing severe hypoxic respiratory failure, particularly those with acute respiratory distress syndrome (ARDS). While protective MV with a low tidal volume (V_T_) is a well-established strategy to mitigate ventilator-induced lung injury (VILI), it may not fully eliminate the risk of lung damage.^1^ Additionally, the compensatory increase in respiratory rate (RR), often required to ensure adequate gas exchange with low V_T_, can increase the mechanical power of ventilation, further contributing to lung injury. Higher RR may cause a greater lung heterogeneity due to a reduction in mean airway pressure and, therefore, in alveolar distension. Moreover, V_T_ and RR, combined according to the formula [(4 × ΔP) + RR], where ΔP is the driving pressure of the respiratory system, are at least informative as mechanical power.^2^ Finally, high inspiratory flow could also contribute to VILI likely relates to locally intensified concentration of stress, in all likelihood because viscoelastic accommodation has insufficient time to dissipate damaging forces when inflation occurs quickly as in the inhomogeneous lungs with ARDS.

These challenges highlight the need for adjunctive strategies such as ultraprotective MV with the use of even lower V_T_. As a result, extracorporeal carbon dioxide removal (ECCO_2_R) is often needed to eliminate the iatrogenic arterial partial pressure of carbon dioxide (PaCO_2_) increased by reduced V_T_ lower than 4 mL/kg of predicted body weight (PBW).^3^ The SUPERNOVA trial^4^ reported a significant reduction in tidal volume and plateau pressure, concomitantly preserving normocapnia and oxygenation after initiation of different ECCO_2_R systems. In the subgroup of patients of Xtravent trial^5^ with a PaO_2_/FiO_2_ ratio of < 150 mmHg, patients undergoing ECCO_2_R plus ultraprotective MV with tidal volumes as low as 3 mL/kg PBW could be weaned off mechanical ventilation faster. A systematic review and meta‐analysis to evaluate the impact of ECCO_2_R on gas exchange and respiratory settings in critically ill adults with respiratory failure, ECCO_2_R effectively reduces PaCO_2_ and acidosis allowing for less invasive ventilation. However, effective use of ECCO_2_R necessitates meticulous control over ventilator settings to avoid hypoxemia and alveolar de-recruitment.^6^ Consequently, esophageal pressure (Pes) monitoring could be a crucial tool in ultraprotective MV using ECCO_2_R to prevent atelectasis and atelectrauma.

The latest ARDS guidelines advise against the routine use of ECCO_2_R for treating ARDS outside the context of randomized clinical trials.^7^ This recommendation reflects concerns over the absence of evidence for mortality benefit, as well as the potential serious side effects such as cerebral hemorrhage and extracranial bleeding. However, improvement of outcome should be pursued not only through the simple reduction of V_T_, but rather in the careful monitoring of intrathoracic pressures. This approach aims to optimize the setting of more protective MV and prevent VILI. In this editorial, we discuss details of ultraprotective ventilation and the importance of Pes monitoring herein.

**Ultraprotective mechanical ventilation:** Although MV with lung protection strategy is the cornerstone in the prevention of VILI in patients with ARDS, tidal hyperinflation and elevated plasma cytokine concentration occur in about 30% of these patients.^1^ Indeed, MV with a low V_T_ (6 mL/kg PBW) may lead to the activation of inflammatory processes and may augment or produce a pulmonary damage that is indistinguishable from that caused by the underlying disease process such as atelectrauma, lung inhomogeneity, surfactant dysfunction, pulmonary edema, and atelectasis. During the ultra-protective ventilation strategy, existing MV requirements are lowered below the standard of care, with V_T_ < 4 mL/kg PBW and plateau pressure (P_plat_) < 25 cmH_2_O to minimize VILI and improve outcomes. By reducing stress (barotrauma) and strain (volutrauma) of the lung, this strategy prevents overdistention, reduces inflammation, and minimizes alveolar damage, improving overall lung protection. However, this strategy can induce iatrogenic hypercapnia and respiratory acidosis, with several detrimental effects, including impairment of right ventricular function, and increased intracranial pressure. Respiratory acidosis may be addressed by decreasing the instrumental dead space, increasing RR, and using ECCO_2_R. The ultra-protective strategy, however, can cause hypoxemia, induced by a drop in mean airway pressure: the likelihood of a lung collapse increases with decreasing ventilation.8 Furthermore, when ventilation is very low, adsorption atelectasis is caused by an oxygen extraction that is higher than the oxygen provided to some alveolar units. The ventilation/perfusion ratio decreases. During ECCO_2_R, the respiratory quotient decreases when the CO_2_ expelled by the natural lungs decreases. The fraction of inspired oxygen (FiO_2_) must be increased to keep the alveolar partial pressure of oxygen (PAO_2_) constant. This can be easily derived from the alveolar gas equation. The lower the FiO_2_, the greater the impact of respiratory quotient on PAO_2_. Finally, to recruit collapsed areas, sufficient pressure must be applied. Despite that, with a Pplat of 25 cmH_2_O, newly developed atelectasis cannot be reopened, and 30–40% of the recruitable lung always remains closed. Positive end-expiratory pressure (PEEP) must be raised by enough to keep the mean airway pressure constant, to preserve the open lung volume, and to increase oxygenation.^8^ These mechanisms confirm the strong association between oxygenation and maintaining appropriate pressures, suggesting that Pes monitoring in these patients is crucial to control respiratory mechanics.

**Esophageal pressure monitoring during ECCO_2_R:** Esophageal Pes monitoring involves placing a nasogastric tube with a balloon that measures Pes swings, which serves as a surrogate for pleural pressure swing. This technique provides a critical insight into transpulmonary pressure (P_L_) (the difference between airway pressure and pleural pressure), a key determinant of VILI.^9^ Clinicians can use esophageal pressure monitoring to tailor protective MV to maintain safe transpulmonary pressure.^10^ In particular Pes allows assessment of partitioned respiratory mechanics and quantification of lung stress, which helps our understanding of the patient’s respiratory physiology and could guide individualization of ventilator settings. Esophageal manometry also allows breathing effort quantification, which could contribute to improving settings during assisted ventilation and weaning from mechanical ventilation. However, VILI is sustained by regional overdistention that can occur during the protective strategy. The goal of the ultraprotective strategy is to provide the lowest possible mechanical power while maintaining the lung as homogenous as possible by prone positioning and adequate PEEP.

**Figure 1:**
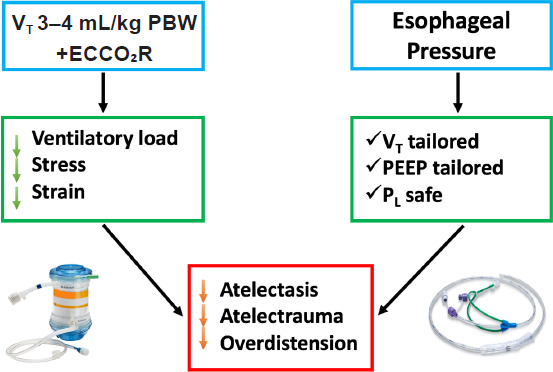
Integration of ECCO_2_R with esophageal pressure monitoring into the management of patients with severe respiratory failure. This framework highlights the combined benefits of using low tidal volume (V_T_; 3–4 mL/kg predicted body weight [PBW]) facilitated by Extracorporeal Carbon Dioxide Removal (ECCO_2_R) and esophageal pressure monitoring to optimize ventilation settings. Key outcomes include reduced ventilatory load, stress, and strain, while ensuring tailored V_T_, positive end-expiratory pressure (PEEP), and safe transpulmonary pressure (P_L_). These strategies collectively aim to prevent atelectasis, atelectrauma, and overdistension. Created with Microsoft PowerPoint.

From a practical standpoint (**Figure 1**), the introduction of ultraprotective MV with ECCO_2_R determines a progressive reduction in transpulmonary driving pressure (the difference between the driving pressure of the respiratory system and the chest wall pressure swing during tidal volume inspired, target < 10–12 cmH_2_O) due to the reduction in V_T_ (3–4 mL/kg PBW) and, simultaneously, a progressive reduction in transpulmonary pressure elastance-derived (total stress as the product of the plateau pressure with the ratio between lung elastance and respiratory system elastance, target < 20 cmH_2_O).^9^ This is associated with a decrease in overdistension areas in the non-dependent zones of the lung and inflammation, particularly in patients with compromised respiratory function, such as low functional residual capacity (small “baby lung”) and reduced respiratory system compliance. In this context, it is particularly important to “tailor” the most appropriate PEEP to achieve a transpulmonary end-expiratory pressure (the difference between PEEP and end-expiratory esophageal pressure) between –2 and +2 cmH_2_O to prevent alveolar collapse (atelectasis), atelectrauma and overdistension^11^ (**Figure 1**). The EPVent trial reported encouraging but limited results for the PEEP titration Pes-guided strategy; regrettably, the EPVent-2 trial did not support these encouraging findings and challenges remain.^11^,^12^ Patients who respond to traditional treatment can be distinguished from those who can benefit from extracorporeal lung assistance (ECMO and ECCO_2_R) by using a method based on the Pes-guided open lung concept, which requires PEEP to be high enough to be transmitted to the collapsed lungs and to overcome chest wall stiffness.

Finally, esophageal pressure monitoring could be very useful in the final stages of treatment with ECCO_2_R when transitioning to assisted MV (i.e., pressure support ventilation) as it allows for the quantification of inspiratory effort in terms of esophageal pressure swing.^9^ During spontaneously breathing or assisted MV for severe ARDS, forced inspiratory efforts may cause an excessive decrease in pleural pressure and an increase in dynamic and static alveolar pressure, which are not evenly distributed throughout the lungs. Keeping P_L_ below the upper limit of 20–25 cmH_2_O should help mitigate harmful inspiratory efforts during active or assisted breathing to prevent patient self-inflicted lung injury, although there are no clear reference values for P_L_.^9^

Continuous esophageal pressure monitoring provides real-time data crucial for timely clinical decision-making. Considering the dynamic ICU environment in which patient conditions can rapidly change, access to accurate and continuous measurements of esophageal pressure could enhance patient safety and improve outcomes, especially during extracorporeal treatment such as ECCO_2_R. Although clinical evidence for Pes-guided mechanical ventilation is yet limited and challenges remain such as the cost of the esophageal catheter and the need for specialized equipment and expertise, technological improvements have made Pes monitoring feasible to become part of bedside respiratory monitoring in selected patients. This should encourage clinicians to develop new clinical studies aimed at identifying optimal and safe Pes-guided targets for the management of the critically ill to improve ICU outcomes.

**Conclusion:** The integration of ECCO_2_R with esophageal pressure monitoring into the management of patients with severe respiratory failure represents a significant opportunity to guide and optimize ventilation settings. By providing clinicians with real-time and accurate data on transpulmonary pressures, these technologies empower individualized ventilation strategies that balance the dual goals of lung protection and adequate gas exchange. Well-designed studies are necessary to test these hypotheses and clarify their clinical utility.
